# Clinical, virological, and antibody profiles of overlapping dengue and chikungunya virus infections in children from southern Colombia

**DOI:** 10.1371/journal.pntd.0013260

**Published:** 2025-09-08

**Authors:** Daniela Polanía-Espinosa, Sebastián Castro-Trujillo, Carlos F. Narváez

**Affiliations:** 1 División de Inmunología, Programa de Medicina, Facultad de Ciencias de la Salud, Universidad Surcolombiana, Neiva, Huila, Colombia; 2 Área de Pediatría, Departamento de Ciencias Clínicas, Facultad de Ciencias de la Salud, Universidad Surcolombiana, Hospital Universitario de Neiva, Neiva, Huila, Colombia; Arizona State University - Tempe Campus: Arizona State University, UNITED STATES OF AMERICA

## Abstract

**Background:**

Dengue and chikungunya are arboviral diseases with overlapping clinical characteristics. Dengue virus (DENV) is endemic in Colombia, and in 2014/2015, the chikungunya virus (CHIKV) caused an epidemic that resulted in over 350,000 cases. Since then, both viruses have been actively co-circulating. The early and accurate identification of pediatric infection caused by DENV or CHIKV is essential for proper medical management. Given that subsequent infections and co-infections with DENV and CHIKV have been reported, virological and immunological factors may influence their clinical outcomes. Here, we analyzed the viremia, antigenemia, and virus-specific antibody responses in hospitalized children suspected of having dengue during the peak of CHIKV infections in Colombia.

**Methods:**

Ninety-one children with a clinical diagnosis of dengue were included in the peak of the CHIKV epidemic (December 2014 to May 2015) at a reference healthcare center in Huila, south of Colombia. Multiplexed RT-qPCR for DENV, CHIKV, and ZIKV was performed, and DENV antigenemia was evaluated using an ELISA for the NS1 antigen. Commercial capture or in-house indirect NS1-based ELISAs were used to assess circulating DENV and CHIKV-IgM and IgG. Clinical and laboratory characteristics were analyzed during hospitalization, and convalescent follow-up was conducted for a fraction of children.

**Results:**

DENV and CHIKV monoinfections were confirmed in 54% and 12% of children, respectively, with the expected virus-specific seroconversion in recovery. Overlapping infections occurred in 22% of the children, while 12% showed no detectable DENV or CHIKV infections. Abdominal pain, vomiting, hepatomegaly, and thrombocytopenia were common findings associated with DENV, while arthralgia and rash characterized CHIKV monoinfections. One fatal secondary DENV-3 monoinfection was registered, and DENV infection dominated the symptoms of overlapping infections without producing different clinical outcomes compared to monoinfections. Thirty-eight percent of children were seropositive for CHIKV-IgG, indicating a significant burden of CHIKV infection in the pediatric population shortly after its introduction in Colombia. The previous virus-specific IgG serostatus did not impact the clinical outcome of the current heterotypic arboviral infection.

**Conclusion:**

The pediatric population in southern Colombia was rapidly exposed to CHIKV infections during the first months following its arrival, with up to 12% of hospitalized children suspected of having dengue experiencing CHIKV monoinfection, supporting that complex and dynamic epidemiological patterns may lead to delayed or missed diagnoses. The overlapping infections of DENV and CHIKV were frequent and did not lead to worse clinical or fatal outcomes.

## Author summary

Dengue and chikungunya are mosquito-borne viruses that often circulate together in the same regions and cause similar symptoms, making it difficult for medical personnel to distinguish between them. In Colombia, the dengue virus (DENV) has been endemic for decades, and the chikungunya virus (CHIKV) emerged in 2014, causing a large epidemic. We studied 91 children hospitalized with suspected dengue during the peak of the chikungunya outbreak in southern Colombia. Using molecular and antibody-based diagnostic tests, we confirmed dengue in more than half of the cases, chikungunya in 12%, and overlapping infections in 22%. We found that dengue dominated the clinical presentation of coinfected children, with abdominal pain, vomiting, and low platelet counts, while chikungunya infections were characterized by joint pain and rash. Importantly, having pre-existing antibodies to either DENV or CHIKV did not worsen the clinical outcome of the current infection. Our findings emphasize the challenges in diagnosing arboviruses in endemic areas and highlight the need for combined diagnostic approaches and continuous surveillance to improve patient care and public health response.

## Introduction

Arboviruses are mosquito-borne diseases that include dengue virus (DENV), chikungunya virus (CHIKV), and Zika virus (ZIKV) infection. DENV is an orthoflavivirus of the family *Flaviviridae*, with four serotypes (DENV1–4) [1]. It is the most widespread arbovirus in the world, with 100 million symptomatic infections reported annually [2]. In Colombia, dengue has been an endemic-epidemic disease for decades, and in 2024, the country reported the second-highest number of cases in South America after Brazil [3,4]. Clinically, the disease is characterized by headache, myalgia, arthralgia, retroocular pain, and maculopapular rash. A fraction of patients, especially the pediatric population with heterologous secondary infections, progress to severe and potentially fatal forms [5].

On the other hand, CHIKV is an arthrogenic alphavirus of the *Togaviridae* family that emerged in the Americas at the end of 2013 and caused 2 million infections, in addition to long-lasting joint sequelae [6]. In Colombia, autochthonous transmission began in September 2014, and 350,000 cases were reported during the epidemic. The virus now maintains an endemic circulation, with sporadic reports of outbreaks in countries such as Brazil and Colombia [7]. Although polyarticular involvement is the most relevant aspect of the disease, CHIKV infection as well as DENV infection share symptoms such as fever, headache, myalgia, rash, and polyarthralgia [8], which significantly complicates their clinical diagnosis.

The interaction between arboviruses and the sequence of infections could influence the clinical manifestations and severity. In fact, *in vitro* studies in human peripheral blood mononuclear cells have shown that simultaneous infection with DENV and CHIKV results in a significant reduction of CHIKV progeny and an increase in DENV production [9]. Natural co-infections by DENV and CHIKV have been reported globally in 2.5% of patients with febrile syndrome, and 37.4% in the 2018 – 2019 period for Colombia, the latter being the highest rate recorded [10]. Although relatively frequent, there is no evidence that DENV-CHIKV co-infections are associated with a more severe clinical outcome or different clinical behavior than monoinfections, although this is still under debate [11,12]

DENV and CHIKV infection induce robust antibody responses. The preexistence of antibodies against non-neutralizing heterologous DENV serotypes is the main immune risk factor for developing severe dengue [13]. Also, previous exposure to ZIKV increases the risk of symptomatic infection with DENV-2 [14]. However, the impact of virus-specific serostatus on the clinical outcome of subsequent DENV and CHIKV infections has been little explored. Although DENV and CHIKV belong to different viral families, some studies report low cross-reactivity between them. Even cross-reactive antibodies against CHIKV can neutralize or increase the burden of DENV infection [15], but their impact on natural infection in co-circulating settings remains unknown.

Few studies have evaluated how epidemiological patterns influence the diagnosis of emerging arboviruses, and how the interaction and sequence of infections between these viruses, as well as the virus-specific immune response, impact the clinical scenario. Here, we analyzed viremia, antigenemia, and virus-specific IgM and IgG antibody responses in children hospitalized with clinical suspicion of dengue during the peak of CHIKV infections in 2014–2015 in southern Colombia, to understand the frequency, clinical characteristics, and serostatus impact of overlapping DENV and CHIKV infections in the pediatric population.

## Materials and methods

### Ethics statement

This study was approved by the Ethics, Bioethics and Research Committee of the Hospital Universitario de Neiva (Approval letter No. 012–012 2015, update No. 24-04-2020). Written informed consent (from parents) and informed assent were obtained from each of the included children (>6 years). All experiments were conducted in accordance with the principles outlined in Resolution 8430 of 1993 of the Colombian government and the Declaration of Helsinki.

### Patients and samples

Ninety-one patients, aged between 1 month and 14 years, with a clinical diagnosis of dengue who were admitted to the pediatric service of the Hospital Universitario Hernando Moncaleano de Neiva in Huila, a reference center for southern Colombia, between December 01, 2014, and May 31, 2015 (the peak of the chikungunya epidemic in Colombia) were included in this study.

**Fig 1:**
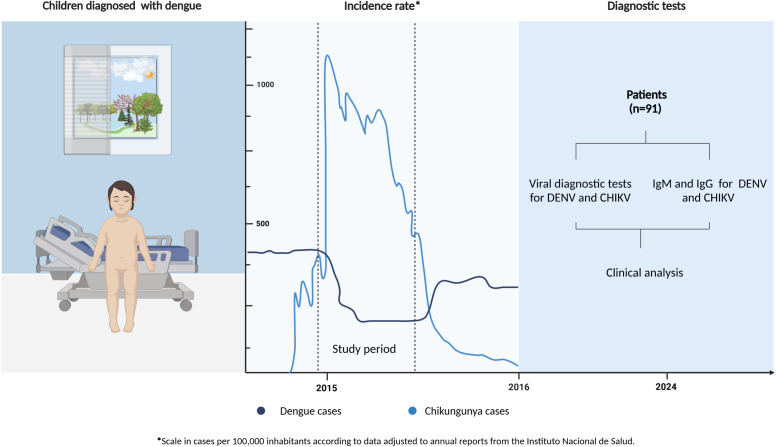
Flow chart of the study design. Ninety-one patients with a clinical diagnosis of dengue hospitalized in the pediatric department of Hospital Universitario de Neiva during the peak of chikungunya cases in Colombia were included. Acute-phase plasma samples (and a fraction of convalescent samples) underwent molecular and serological assays to identify the infecting arbovirus, circulating NS1 (for DENV), and virus-specific IgM/IgG responses. Clinical follow-up was performed during hospitalization. This figure was created with BioRender.com (license ID JB27OLTWT5).

Peripheral venous whole blood sample of 2 mL (or a volume adjusted to the weight of the children) was collected in tubes containing K-ethylenediaminetetraacetic acid (EDTA-K^++^, catalog No 368171, BD vacutainer), during the acute (1–5 days from fever onset) and convalescence phase (15–33 days after hospital discharge) in a fraction of patients (n = 34). Samples were transported at room temperature and then centrifuged at 200 *× g* for 10 minutes. The resulting plasma was stored at -80°C until analysis. Diagnosis, classification, and treatment of patients were performed according to the recommendations outlined in the Guidelines for the management of patients with dengue and the revised World Health Organization (WHO) 2009 classification [16]. The study design is shown in Fig 1.

### Nucleic acid amplification tests (NAAT) for DENV and CHIKV

Automated purified RNA was isolated using the KingFisher Flex instrument (Thermo Scientific, catalog No. 5400610, Singapore) and the commercial MagMax Viral/Pathogen II kit (Thermo Applied Biosystems, catalog No. A48383), with a previously reported protocol [17]. The genome of DENV and CHIKV was identified by real-time reverse transcriptase quantitative polymerase chain reaction (RT-qPCR) using the commercial TaqMan Arbovirus Triplex kit 0.1mL (Thermo Applied Biosystems, catalog No. A31747, kindly donated by Equipos y Laboratorio de Colombia S.A.S) on the Quant Studio 5 instrument (Thermo Applied Biosystems, catalog No. A28134) following the manufacturer’s instructions.

### Detection of circulating NS1

DENV-specific NS1 in plasma was assessed by ELISA kit (Panbio Dengue Early, catalog No. 01P4P. Standard Diagnostic Inc., Gyeonggi-do, Korea) or DENV Detect NS1 ELISA (InBios International Inc., Seattle, WA, catalog No. DNS1–1), applied according to each manufacturer’s recommendations. Briefly, 100 µl/well of half-diluted plasma (diluted with sample dilution buffer) were added to the pre-coated plate and incubated for 1 hour at 37 °C. The plate was washed six times with 300 µl of wash buffer, and 150 µl of horseradish peroxidase-conjugated polyclonal antibody were added and incubated for 30 minutes at 37 °C. Then plate was washed and 100 µl of TMB substrate were added. The reaction was stopped with 50 µl of 1 N H₂SO₄. Absorbance was measured at 450 nm using a Varioskan LUX automated multimode microplate reader (Thermo Scientific, catalog No. N16706, Singapore). Positive, negative controls, and cutoff wells provided for the kit were included in all assays and evaluated in duplicate.

### Detection of IgM and IgG specific for DENV and CHIKV

DENV-specific IgM and IgG levels were assessed by an indirect ELISA based on a mixture of recombinant NS1 proteins (rNS1) from the four DENV serotypes 1–4, previously optimized and validated by us [18,19]. Immulon 4 HBX 96-well plates (ThermoScientific, catalog No. 3855) were coated overnight at 4°C with 50 μL of a mixture of rNS1 (all from The Native Antigen Company, Kidlongton, UK) of DENV-1 (Nauru/Western Pacific/1974), DENV-2 (Thailand/16681/84), DENV-3 (Sri Lanka D3/H/IMTSSA/SRI/2000/1266) and DENV-4 (Dominica/814669/1981) at a final concentration of 2 μg/mL (0.5 ug/mL of each serotype) for IgM-NS1 or 32 ng/mL for IgG-NS1 (8 ng each rNS1 serotype). Wells were blocked for 1h with 150 μL of 5% Blotto (skim milk powder, Chem Cruz, catalog No SC-2325 in 1X phosphate buffered saline plus 0.1% Tween 20). The contents were removed and 50 μL of plasma diluted 1/100 for IgM-NS1 or 1/200 for IgG-NS1 in 2.5% blotto was deposited in the wells. After 4 washes with phosphate buffered saline, 0.1% Tween-20 (wash buffer), 50 μL of biotin-labeled goat anti-human IgM or IgG (Seracare-KPL, cat: 16103, Naryland, USA) were added at a concentration of 0.5 μg/mL, followed by incubation for 1h at 37°C. They were then incubated with 50 μL of 0.5 μg/mL streptavidin-peroxidase (Seracare-KPL, catalog No. 5270–0029, Milford, MA, USA) for 1h at 37°C. Finally, 50 μL of tetramethylbenzidine A and B components (Seracare-KPL, catalog No. 51200049 and 51200038) were added. The reaction was stopped with 50 μL of H_2_SO_4_ 2M (Merck, Darmstadt, Germany; catalog No. 112080), and the wells were read at 450 nm on a Varioskan Lux automated multimode microplate reader (ThermoScientific, catalog No. N16706, Singapore). Additionally, for a subset of patients (n = 11), IgG-DENV levels were assessed by a commercially available capture ELISA kit (Virion/Serion IgG DENV, catalog No. ESR114G), following the manufacturer’s suggestions. Plasma from unexposed and patients with confirmed DENV infection in the convalescent phase (30 days after symptom onset) were entered as controls on all plates. A < 10% variability in the optical densities at 450nm of the controls from plate to plate was obtained.

Plasma CHIKV-IgM and CHIKV-IgG levels were assessed using commercially available ELISA kits approved for human diagnostic use: NovaTec Chikungunya Virus IgM μ-capture, catalog No. CHIM0590; InBios CHIKVjj Detect IgM ELISA, catalog No. 900186–08; Nova Tec Chikungunya Virus IgG capture, catalog No. CHIG0590, respectively, following the manufacturers’ instructions. For example, for the CHIKV-IgG evaluation, 50 µl of diluted plasma (1/100) in 1 mL of dilution solution were added and incubated for one hour at 37 °C in the pre-coated plate. Wells were washed thrice with 300 µl of wash buffer, and 50 µl of chikungunya antigen were added and incubated for 30 minutes at room temperature. After washing, wells were incubated with 50 µl of biotinylated antibody for 30 minutes at room temperature. Subsequently, wells were washed, and 50 µl of horseradish peroxidase–labeled streptavidin were added. The plate was incubated for 30 minutes at room temperature. Finally, wells were washed, and 100 µl of tetramethylbenzidine (TMB) were added. The reaction was stopped with 100 µl of H₂SO₄ (0.2 M). Absorbance was measured at 450 nm using a Varioskan LUX automated multimode reader (Thermo Scientific, catalog No. N16706).

### Case definitions

Confirmed DENV infection was defined as the presence of any of the following: detection of viral RNA by RT-qPCR, circulating NS1, or the detection of plasma DENV-IgM, the latter two determined by ELISA. Secondary infection was defined as any confirmed DENV infection with DENV-IgG detected by ELISA performed on a plasma sample taken within the first 5 days of symptoms. Confirmed CHIKV infection was defined as the detection of viral RNA in serum using multiplexed RT-qPCR or the detection of circulating CHIKV-IgM. Overlapping infection corresponded to pediatric cases that simultaneously presented at least one of the markers used to define confirmed DENV and CHIKV infections. Prior exposure to CHIKV was confirmed by the detection of circulating virus-specific IgG antibodies using the commercial Nova Tec Chikungunya Virus IgG capture kit.

### Statistical analysis

Statistical analysis was performed with GraphPad Prism version 8.0 software for Microsoft (La Jolla, CA, United States). The results of quantitative variables are presented as medians and ranges. The Chi-square and Fisher tests were used to compare the frequency of categorical variables. The Mann-Whitney and Wilcoxon tests were used to compare continuous variables between two independent and dependent groups, respectively. The Kruskal-Wallis test was used to compare continuous variables in more than two independent groups, and differences between each of the groups were assessed by the Bonferroni-adjusted posttest. A p-value <0.05 was considered significant.

## Results

### Confirmation of DENV and CHIKV infection

Accurate diagnosis is essential for an adequate medical approach. Initially, we aimed to confirm the diagnosis of dengue using both virological and serological methods. Seventy-six percent of the children had detectable viremia, antigenemia, or DENV-IgM, ensuring the diagnosis (S1 Table). Most of the dengue cases were identified by virological and serological methods, confirming that the combination of methods is a useful tool in the diagnosis of dengue in hyperendemic areas [20].

The application of diagnostic methods for dengue allowed classification of children with and without acute DENV infection (Table 1). Children with confirmed infection were older, and vomiting, abdominal pain, and hepatomegaly were the most frequent symptoms. In contrast to children with dengue, arthralgia and rash (with a trend) predominated in DENV-negative children. In line with previous reports [21,22], marked neutropenia, thrombocytopenia, and increased AST characterized children with confirmed DENV infection, which supports the good performance of the diagnostic methods used to properly identify children with and without dengue. Additionally, one-fourth of the children evaluated developed severe dengue, making the cohort useful for analyzing factors associated with clinical severity (Table 1).

**Table 1:** Clinical and laboratory features of children with or without DENV infection included in the study.

Characteristics	Dengue positive (n = 66)^*^	Dengue negative (n = 19)	*p-value*
**Clinical features**			
Age (months), median (range)	91.5 (4 – 179)	36 (1 – 116)	**0.0030**
Male/Female (%)	38/28 (57.5/42.4)	10/9 (52.6/47.3)	0.4464
Dengue classification, n (%)			
DWS	51 (77.2)	-	-
SD	15 (22.7)	-	-
Day after fever onset, median (range)	5 (1 – 8)	4 (1 – 10)	0.9764
Vomiting, n (%)	41 (62.1)	7 (36.8)	0.1075
Abdominal pain, n (%)	36 (54.5)	3 (15.7)	**0.0036**
Epistaxis, n (%)	8 (11.5)	3 (15.7)	0.6972
Hepatomegaly, n (%)	41 (62.1)	4 (21)	**0.0019**
Rash, n (%)	12 (18.1)	7 (36.8)	0.1175
Arthralgia, n (%)	8 (12.2)	7 (36.8)	**0.0348**
**Laboratory parameters, median (range)**			
Hemoglobin (g/dL)	12.9 (9.6 – 17)	11.2 (9.2 – 13.4)	**<0.0001**
Leukocytes (cells/mm^3^)	5,600 (1,900 – 14,690)	5,800 (2,400 – 10,100)	0.8322
Neutrophils (cells/mm^3^)	1,759 (350 – 8,282)	2,600 (1,000 – 8,057)	**0.0187**
Platelets (cells/mm^3^)	56,000 (11,000 – 339,000)	143,000 (31,000 – 499,000)	**<0.0001**
AST (U/L)	146.5 (24 – 2,454)	59.9 (15.4 – 743)	**0.0010**

Categorical and continuous variables were analyzed using Fisher’s exact and Mann-Whitney U tests. P < 0.05 was considered significant.

*The number of patients differs because six patients lack clinical information. DWS, dengue with warning signs; SD, severe dengue; AST, aspartate aminotransferase.

Although suspected of DENV infection, the children studied here were included during the peak of CHIKV infections in Colombia. Therefore, we hypothesized that CHIKV infections could also occur in this pediatric cohort and, as for dengue, molecular and serological tests were applied to identify CHIKV infections. As shown in S1 Table, unlike what was found for DENV, most CHIKV infections were identified by the presence of CHIKV-IgM, supporting its usefulness in the diagnosis of arboviruses in endemic areas, followed by RT-qPCR, which demonstrated active infection in 4 cases (S1 Table). Supporting the specificity of the RT-qPCR multiplex assay used here, ZIKV infections were not detected in any case, as the virus did not arrive in Colombia until October 2015.

Thus, a relatively high frequency of chikungunya cases was identified in hospitalized children with a clinical diagnosis of dengue, demonstrating the significant impact of epidemiological patterns of arboviruses in the pediatric diagnosis of these diseases.

### Characteristics of monoinfections and overlapping infections with DENV and CHIKV

The application of the panel of molecular and serological diagnostic methods for DENV and CHIKV allowed the identification of DENV monoinfections, monoinfections with CHIKV, overlapping infections with DENV and CHIKV, and other febrile illnesses (OFI), with frequencies of 54%, 12%, 22%, and 12%. In addition, the assessment of plasma DENV-IgG within the first 5 days of symptoms allowed the classification of dengue cases into primary or secondary infections (Table 2).

**Table 2:** Clinical and laboratory features of children after diagnosis confirmation.

Characteristics	DENV monoinfections (n = 46)		CHIKV monoinfections (n = 10)	Overlapping infections (n = 20)	OFI (n = 9)	*p-value*
	Primary (n = 14)	Secondary (n = 32)				
**Clinical features**						
Age (months), median (range)	45.5 (4 – 179)	99.5 (4 – 168)	40 (1 – 116)	100 (32 – 172)	36 (8 – 103)	**0.0091**
Male/Female (%)	8/6 (57.1/42.8)	17/15 (53.1/46.8)	6/4 (60/40)	13/7 (65/35)	6/3 (66.6/33.3)	0.6113
Dengue classification, n (%)						
DWS	11 (78.5)	26 (81.2)	10 (100)	16 (80)	3 (33.3)	0.5771
SD	3 (21.4)	8 (18.7)	0 (0)	4 (20)	1 (11.1)	-
Day after fever onset, median (range)	4.5 (2 – 8)	4 (1 – 6)	5 (1 – 8)	5 (2 – 7)	4 (1 – 10)	0.7781
Vomiting, n (%)	10 (71.4)	18 (56.2)	5 (50)	13 (65)	2 (22.2)	0.1757
Abdominal pain, n (%)	5 (35.7)	17 (53.1)	2 (20)	15 (75)	1 (11.1)	**0.0046**
Epistaxis, n (%)	1 (7.1)	4 (12.5)	2 (20)	3 (15)	1 (11.1)	0.9147
Hepatomegaly, n (%)	9 (64.2)	19 (59.3)	3 (30)	13 (65)	1 (11.1)	**0.0282**
Rash, n (%)	4 (28.5)	5 (15.6)	6 (60)	3 (15)	2 (22.2)	0.4163
Arthralgia, n (%)	3 (21.4)	5 (15.6)	5 (50)	2 (10)	2 (22.2)	0.2598
**Laboratory parameters, median (range)**						
Hemoglobin (g/dL)	12.1 (9.6 – 16.2)	12.9 (10.7 – 17)	11.4 (10 – 13.4)	13.4 (11.3 – 15.2)	11 (9.2 – 12.9)	**0.0001**
Leukocytes (cells/mm^3^)	7,000 (2,700 – 10,200)	4,950 (1,900 – 14,690)	5,995 (2,400 – 9,490)	5,450 (3,200 – 14,300)	5,000 (3,700 – 10,100)	0.8759
Neutrophils (cells/mm^3^)	1,700 (600 – 8,282)	2,050 (350 – 6,700)	2,950 (1,000 – 8,057)	1,606 (600 – 6,635)	2,500 (1,117 – 7,700)	0.1307
Platelets (cells/mm^3^)	71,500 (23,000 – 294,000)	47,500 (11,000 – 339,000)	143,500 (31,000 – 345,000)	53,450 (21,000 – 214,000)	143,000 (48,000 – 499,000)	**0.0001**
AST (U/L)	117 (43.3 – 724)	148 (24 – 2,454)	56.3 (37.5 – 162.7)	143.3 (76.1 – 605.9)	63.9 (15.4 – 743.0)	**0.0160**

Data are median (range) or n (%). Categorical and continuous variables were analyzed using the Chi-square and Kruskal-Wallis (with post hoc adjustment) tests. P < 0.05 was considered significant.

*The number of patients differs because six patients do not have clinical information. OFI: Other febrile illness. DWS, dengue with warning signs; SD, severe dengue; AST, aspartate aminotransferase.

Consistent with an endemic circulation pattern and the recent introduction of a new arbovirus in the pediatric population, primary DENV and CHIKV monoinfections occurred more frequently in younger children, in contrast to secondary DENV monoinfections and overlapping infections (Table 2). We then analyzed the clinical and laboratory data of the groups to determine differences in outcome between monoinfections and overlapping infections. Consistent with the application of hospitalization criteria in dengue patients with warning signs, abdominal pain and hepatomegaly were frequent clinical markers of all groups with DENV infection, regardless of whether it was monoinfection or overlapping infection with CHIKV (Table 1). Also, significant hemoconcentration, thrombocytopenia, and AST elevation were only found in these groups, demonstrating a clear clinical and laboratory dominance of DENV infection over CHIKV in overlapping infections (Table 2). This indicates a likely recent CHIKV exposure but acute DENV infection, given that all overlapping infections included only CHIKV-IgM positive results. In contrast, children with CHIKV monoinfections frequently presented rash, arthralgia, and much less drastic changes in platelet and liver enzyme values, laboratory features comparable to those found in the OFI group (Table 2). A fatal case of secondary DENV-3 monoinfection was reported in a 10-year-old patient, attributed to hypovolemic and cardiogenic shock. During the 5-day hospitalization, the patient presented with severe thrombocytopenia, hemoconcentration, and persistently elevated transaminase levels. Myocarditis was suspected based on positive troponin findings. Notably, all four DENV serotypes were reported to co-circulate throughout the country in 2015.

Overall, these results demonstrate the early and high burden of exposure to co-circulating arboviruses among the pediatric population of southern Colombia and confirm helpful clinical and laboratory differences for the diagnosis of dengue and chikungunya.

### Acute and convalescent arbovirus-specific antibody response

**Fig 2:**
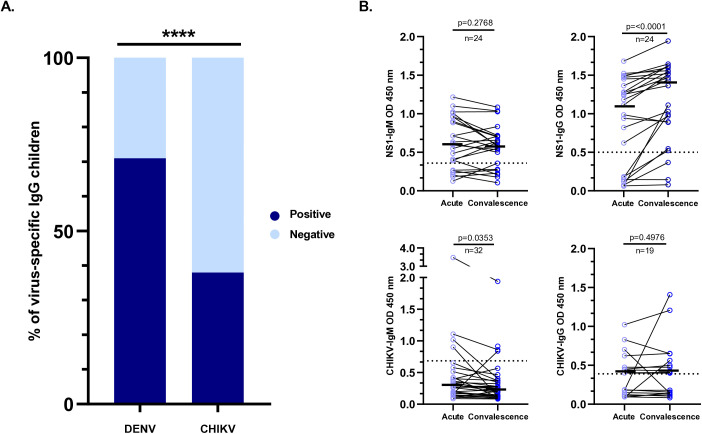
(A) DENV-IgG and CHIKV-IgG serostatus during the acute phase. Bars show the percentage of seropositive and seronegative individuals; labels on bars indicate % and n (Chi-square test, p < 0.0001). (B) Seroconversion of DENV-/CHIKV-IgM and IgG in paired acute/convalescent samples; shows paired n and the statistical test used for paired comparisons if performed.

Plasma arbovirus-specific IgM and IgG are immune markers of current or past infection, offering commonly used tools to support diagnosis and establish the exposure burden of a population to these pathogens. Therefore, we assessed DENV- and CHIKV-specific IgM and IgG, and in a fraction of the children, this assessment was performed in pairs during their convalescence. After initial assessment of plasma DENV and CHIKV-IgG, and in agreement with previous studies demonstrating early and continuous exposure to DENV in hyperendemic areas of Colombia and the high frequency of secondary infections in hospitalized children with dengue [19,23], 70% of the children studied were seropositive for DENV-IgG (Fig 2A). Additionally, and in support of the rapid and efficient introduction and spread of CHIKV in the pediatric population of southern Colombia, 38% of the children tested in the acute phase were seropositive for CHIKV-IgG (Fig 2A).

An increase in plasma levels of virus-specific IgM and IgG between the acute and convalescent phases is one of the most reliable tools to confirm infection. DENV and CHIKV-IgM and IgG levels were assessed in the acute and convalescent phases in a fraction of the children. A clear seroconversion was found in DENV-IgG, but not in DENV-IgM, a result explained by the low magnitude of the IgM response in secondary infections suffered by most hospitalized children (Fig 2B). In contrast, clear changes in CHIKV-IgM and IgG levels were not observed, probably because of the lower number of chikungunya cases included in the cohort (Fig 2B).

### Relationship between the arboviral-specific IgG serostatus and clinical outcome

The pre-existence of non-neutralizing heterologous antibodies and the sequence of infections are critical factors that determine the clinical outcome of exposure to co-circulating arboviruses, such as DENV and ZIKV [24]. Although DENV and CHIKV belong to different viral families, previous animal and human studies have shown varying degrees of neutralizing and non-neutralizing IgG antibody cross-reactivity [15]. Therefore, we evaluated whether serostatus, defined as the preexistence of virus-specific IgG, modified the clinical outcome of current infections with DENV and CHIKV. Children seropositive and seronegative for CHIKV-IgG with current DENV infection, in addition to children seropositive for DENV-IgG and current CHIKV infection, were clinically compared. Because of the high seroprevalence for IgG-DENV in hyperendemic areas (Fig 2A), the pediatric group seronegative for IgG-DENV with current CHKV infection was unavailable. Despite clear differences in baseline virus-specific IgG before current heterotypic infection, no clinical differences or differences in laboratory markers assessed, other than the frequency of children with severe forms of infection, were observed between the groups (Table 3). An analysis of the relative frequency (%) of severe cases between the groups in fact showed that current CHIKV infection in DENV-IgG seropositive children was not associated with severe disease requiring ICU management (Table 3). In summary, the data indicate that the preexistence of virus-specific IgG does not modify the clinical outcome of current heterologous DENV or CHIKV infections, detracting from the practical importance of ADE within the pathophysiologic process during these specific infections.

**Table 3:** Impact of the heterologous arbovirus-specific serostatus on the DENV and CHIKV infection clinical outcome.

Characteristics	CHIKV-IgG seronegative with DENV infection (n = 13)	CHIKV-IgG seropositive with DENV infection (n = 7)	DENV-IgG seropositive with CHIKV infection (n = 7)	*p-value*
**Clinical features**				
Age (months), median (range)	115 (4 – 168)	108 (12 – 116)	36 (1 – 116)	0.1768
Male/Female (%)	4/9 (30.7/69.2)	5/2 (71.4/28.5)	4/3 (57.1/42.8)	0.1902
Dengue classification, n (%)				
DWS	9 (69.2)	5 (71.4)	7 (100) ^*^	0.2576
SD	4 (30.7)	2 (28.5)	0 (0)	-
Day after fever onset, median (range)	4 (2 – 6)	4 (3 – 8)	4 (1 – 6)	0.8509
Vomiting, n (%)	7 (43.7)	5 (71.4)	5 (71.4)	0.6397
Abdominal pain, n (%)	7 (43.7)	4 (57.1)	1 (14.2)	0.2268
Epistaxis, n (%)	1 (7.9)	2 (28.5)	2 (28.5)	0.3777
Hepatomegaly, n (%)	1 (7.9)	3 (42.8)	3 (42.8)	0.1142
Rash, n (%)	4 (30.7)	2 (28.5)	4 (57.1)	0.2905
Arthralgia, n (%)	5 (38.4)	0 (0)	3 (42.8)	0.1609
**Laboratory parameters, median (range)**				
Hemoglobin (g/dL)	12.4 (9.6 – 17)	12.7 (10.7 – 15.1)	11.5 (10 – 13.4)	0.2049
Leukocytes (cells/mm^3^)	5,700 (1,900 – 10,400)	4,900 (2,800 – 14,690)	5,700 (2,400 – 7,500)	0.7380
Neutrophils (cells/mm^3^)	1,700 (600 – 8,282)	2,000 (1200 – 4,800)	2,500 (1,000 – 4,800)	0.5909
Platelets (cells/mm^3^)	43,000 (21,000 – 222,000)	56,000 (33,000 – 100,000)	130,000 (31,000 – 345,000)	0.1044
AST (U/L)	138.8 (70 – 2,018)	145 (77.4 – 169)	55 (37.5 – 162.7)	0.0966

Categorical and continuous variables were analyzed using the Fisher and the Kruskal-Wallis (and post hoc) tests, respectively. P < 0.05 was considered significant.

*Clinical diagnosis. DWS, dengue with warning signs; SD, severe dengue; AST, aspartate aminotransferase.

## Discussion

The identification of arboviruses responsible for clinically similar diseases is a critical aspect of the medical approach, further complicated by the highly dynamic circulation patterns of these viruses. Much of the clinical outcome of arbovirus infection is determined by viral and immunological factors such as viral load and specific antibody response. Here, we analyze the frequency and clinical, virological, and immunological characteristics of CHIKV infections during its epidemic in children hospitalized for dengue in southern Colombia, seeking to establish the impact on diagnosis and clinical outcome. Although NAATs are considered the cornerstone of arbovirosis diagnosis, serious limitations such as the short viraemia window, high cost, and use of complex instruments have favored serological assays as a useful diagnostic tool. In the case of DENV infection, the combination of RT-qPCR, NS1, and plasma IgM can detect at least 90% of dengue cases evaluated within 1–10 days of symptom onset [25]. Similar to dengue diagnosis, NAATs, together with IgM-CHIKV detection, reliably identified chikungunya cases [26], also providing basic information on the humoral immune response.

Consistent with previous studies showing that in hyperendemic regions DENV is the predominant infectious agent even during the introduction of new arboviruses [27], cases of DENV monoinfection, followed by DENV-CHIKV overlapping infections, and, to a lesser extent, CHIKV monoinfections were identified. Significant evidence has demonstrated the frequent occurrence of DENV-CHIKV co-infections. Twenty-two percent of the cases evaluated here showed markers of overlapping DENV and CHIKV infection in line with the high frequency of co-infections reported in Colombia [10]. In a significant fraction of cases considered dengue, the diagnosis of chikungunya was confirmed, and this delayed or misdiagnosis has important implications for the medical approach. Co-infection with CHIKV in a child with dengue may mask warning signs, delaying appropriate management. On the other hand, although the mainstay of dengue treatment is intravenous fluid therapy, its application in a child with chikungunya could cause serious complications such as pulmonary edema. Currently, the WHO-PAHO guidelines for the detection and surveillance of co-circulating arboviruses recommend searching for other arboviruses in patients with NAAT-negative for dengue, ruling out the active search for co-infections [28]. However, during epidemics due to the introduction of emerging arboviruses, the search for co-infections could complement the diagnosis and surveillance of public health services. Overall, these results highlight the challenges in diagnostic accuracy in cocirculation settings and underscore the need for robust and continuous virological surveillance.

In agreement with the clinical features, vomiting, abdominal pain, and hepatomegaly for dengue, as well as rash and arthralgia for CHIKV, were key symptoms to identify monoinfections by these viruses. In addition, thrombocytopenia and elevated AST are consolidated as valuable laboratory tools for distinguishing the two infections. In contrast, the features of DENV infection, including thrombocytopenia and elevated AST, largely dominated the clinical and laboratory manifestations of DENV-CHIKV overlapping infections, which also did not show greater severity relative to monoinfections, as previously noted [29–31]. A sequential infection by CHIKV and subsequently by DENV, the latter responsible for the current picture, may be a possibility, since the genomes of the two viruses could not be detected in any of the children studied. However, simultaneous detection of DENV and CHIKV RNA in plasma is rare due to the substantial interaction between the two viruses during co-infection [32]. Consistent with our results, *in vitro* co-infection of human PBMC with DENV and CHIKV significantly suppressed CHIKV replication and increased secretion of IL-6 and TNF-α, two proinflammatory cytokines highly associated with dengue pathogenesis [9]. Additionally, the pattern of circulating cytokines in coinfected patients, including IL-2, IL-8, IFN-α, and IFN-γ, was comparable to DENV monoinfections, but not to CHIKV [32]. The sequence of infection also appears to be a determinant in the outcome of co-infection, since initial infection with CHIKV *in vitro* significantly suppressed subsequent DENV infection, although this is not observed when the sequence of infection is changed [33]. In summary, the clinical outcome of DENV and CHIKV co-infections may result from a combination of factors, including cell type, host, infection sequence, and immune status, which would explain the still inconclusive results of the studies.

Epidemiological and environmental factors, as well as previous exposure to other arboviruses and vector density, influence the variable seroprevalence of CHIKV-IgG in Colombia, which has been estimated to range from 3% to 82% [34,35]. Consistently, we report that during the first 4 months since the introduction of CHIKV to the country, 38% of the children evaluated were positive for CHIKV-IgG, confirming the very high and early exposure of the pediatric population to arboviruses, revealing the complex situation and the need for timely interventions.

For DENV serotypes and even for other orthoflaviviruses such as ZIKV, the preexistence of non-neutralizing heterotypic antibodies that favor viral entry into immune cells expressing FcγR is one of the main immune factors associated with severity. Despite belonging to different families and sharing only 5% homology between their genomes [36], some cross-reactivity of human and murine immune sera, as well as monoclonal antibodies against DENV and CHIKV, has been demonstrated to be capable of mediating antibody-dependent enhancement (ADE) *in vitro* and neutralization *in vivo* [14,15]*.* Consistent with previous studies that ruled out a role of serostatus in the clinical outcome of the current infection [30,31], our results show that the severity of DENV infection was independent of the presence of plasma CHIKV-IgG, thereby ruling out a clinical impact of the reported cross-reactivity. Studies with a larger number of highly serologically characterized patients are needed to establish this relationship, particularly the effect of DENV-IgG on current CHIKV infection, a condition that could not be addressed in the study because of the unavailability of DENV-seronegative children (due to the high burden of circulation in the region) with current CHIKV infection.

Limitations of the study include the small cohort size, the lack of antigenemia assessment to confirm CHIKV infection, and the limited longitudinal follow-up, particularly in terms of osteoarticular sequelae. All these factors may affect the ability of the study to identify small clinical and laboratory differences between monoinfected and co-infected individuals.

In summary, a significant fraction of children hospitalized for dengue during the chikungunya epidemic in Colombia had CHIKV overlapping or monoinfections, a fact that demonstrates the high impact of the dynamic epidemiological patterns of these viruses on the proper diagnosis and medical approach. We found no evidence that co-infections or the preexistence of heterotypic virus-specific antibodies between DENV and CHIKV were associated with a worse clinical outcome. These results support a complex situation with serious epidemiological, medical, and public health implications that require the implementation of interventions such as strengthening diagnostic capabilities, clear algorithms for the management of children with febrile syndrome, vector control, community education, and evaluation of the mass application of available vaccines for DENV and CHIKV.

## Conclusions

In our pediatric cohort hospitalized with a diagnosis of dengue during the epidemic peak of the CHIKV infections, 54% and 12% of the children had confirmed DENV and CHIKV monoinfections, respectively. In comparison, 22% presented overlapping infections, and no evidence of arbovirus infection was found in 12% of cases. The introduction of a newly circulating arbovirus into a dengue-endemic region led to frequent misdiagnoses and complex interaction patterns, including overlapping and sequential infections. Notably, overlapping infections or pre-existing heterotypic virus-specific IgG antibodies did not affect clinical severity or outcomes. This highlighted the need to strengthen the differential diagnosis of arboviruses in co-circulating scenarios to improve clinical care and epidemiological surveillance.

## Supporting information

S1 Table Profile of the diagnostic tests for DENV and CHIKV infections.
(DOCX)
