# Supplementary material for: Clinical, virological, and antibody profiles of overlapping dengue and chikungunya virus infections in children from southern Colombia
Source: PLoS Negl Trop Dis. 2025 Sep 8;19(9):e0013260. doi: 10.1371/journal.pntd.0013260 (PMC12435647; doi:10.1371/journal.pntd.0013260)
Supplement: S1 Table — Profile of the diagnostic tests for DENV and CHIKV infections. (DOCX) [file pntd.0013260.s001.docx]

**SUPPORTING INFORMATION**

| Supp Table 1. Profile of diagnostic tests for DENV and CHIKV infections. | | |
| --- | --- | --- |
| Profile test | **DENV, n (%)** | **CHIKV, n (%)** |
| IgM only | 16 (17.5) | 26 (28.5) |
| Virological (DENV RT - qPCR, NS1), (CHIKV RT - qPCR) | 8 (8.7) | 4 (4.3) |
| IgM + virological | 45 (49.4) | 1 (1.09) |
| Negative | 22 (24.1) | 60 (65.9) |

Markers for acute DENV and CHIKV infection; IgM only: patients with virus-specific IgM only positive, with RT-qPCR and DENV-NS1 negative; Virological (RT-qPCR, DENV-NS1): patients with virus-specific IgM negative and RT-qPCR and/or DENV-NS1 positive; IgM + virological (RT-qPCR, DENV-NS1): patients with virus-specific IgM positive and RT-qPCR and/or DENV-NS1 positive; Negative: patients negative for the active infection markers applied in the study.
